# The Hidden Burden of Undiagnosed ADHD among Medical Students in Pakistan: A Cross-Sectional Survey of Self-Reported Symptoms

**DOI:** 10.1192/j.eurpsy.2025.1854

**Published:** 2025-08-26

**Authors:** Z. U. Nisa

**Affiliations:** 1 Psychiatry, Liaquat National Hospital, Karachi, Pakistan

## Abstract

**Introduction:**

Attention-deficit/ hyperactivity disorder (ADHA) is recognized as a major public health issue, characterized as a persistent neurodevelopmental disorder that presents challenges in various aspects of life, often continuing into adulthood and frequently going undiagnosed.

**Objectives:**

This study aimed to explore the prevalence, types, participants knowledge and perceptions and demographic determinants of undiagnosed adult ADHD among undergraduate medical students in Pakistan.

**Methods:**

This study conducted from July 2023 to December 2023. A nationwide cross-sectional study enrolled 342 undergraduate medical students who met the selection criteria. Data was collected through an online self-administered survey of three main parts, utilizing the WHO 18 questions Adult ADHD Self-Report Scale, Version 1.1 (ASRS-v1.1), to assess adult ADHD symptoms. Data analysis was carried out using SPSS ( version 26.0).

**Results:**

Out of 342 participants, 119 medical students, or 34.8%, were found to have adult ADHD. The most prevalent presentation was inattentive dominance, observed in 86 students (72.3%), followed by mixed dominance in 20 students (16.8%), and hyperactive dominance in 13 students (10.9%). There was a statistically significant (p < 0.05) association between individuals screening positive for adult ADHD and the presence of co-occurring psychological disorders (e.g., anxiety, depression) and a family history of psychiatric disorders (e.g., ADHD, generalized anxiety disorder, bipolar disorder). Additionally, these individuals believed that adults with adult ADHD could lead a normal life despite their condition. The type of ADHD was significantly associated with the use of medications for psychological disorders, with a notably higher usage among hyperactive dominants (5, 71.4%), and a significantly higher family history of GAD among mixed dominants (2, 10.0%).

**Conclusions:**

This study uncovers a significant prevalence of undiagnosed adult ADHD and an inattentive dominance among medical students in Pakistan, highlighting the need for enhanced awareness and screening. These findings underscore the critical necessity for the implementation of ADHD screening programs.

**Disclosure of Interest:**

None Declared

